# Rotavirus Vaccination Protects Against Diabetes Mellitus Type 1 in Children in Developed Countries: A Systematic Review and Meta-Analysis

**DOI:** 10.3390/vaccines13010050

**Published:** 2025-01-09

**Authors:** Chrysoula Kosmeri, Achilleas Klapas, Nikolas Evripidou, Evanthia Kantza, Anastasios Serbis, Ekaterini Siomou, Fani Ladomenou

**Affiliations:** 1 Department of Pediatrics, University Hospital of Ioannina, 45500 Ioannina, Greece; chrisa.kosmeri@gmail.com (C.K.); kantzaevina@gmail.com (E.K.); aserbis@uoi.gr (A.S.); eksiomou@yahoo.gr (E.S.); 2 Medical School, University of Ioannina, Stavrou Niarchou Street, 45500 Ioannina, Greece; md06846@uoi.gr (A.K.); md06816@uoi.gr (N.E.)

**Keywords:** rotavirus vaccine, type 1 diabetes mellitus, vaccination, child

## Abstract

**Background**: The etiology of type 1 diabetes (T1D) remains an area of active research, with genetic and environmental factors being investigated. This meta-analysis aimed to determine if rotavirus vaccination influences the onset of T1D in children. **Methods**: Following PRISMA 2020 guidelines, two researchers independently searched multiple databases, including PubMed and Google Scholar, for studies published in English from 2006 to September 2024. They used the search terms “rotavirus vaccination” and “type 1 diabetes”, and assessed study quality using the ROBINS-E tool. The analysis pooled hazard ratios (HRs) from selected studies using a fixed-effects model, with statistical significance set at *p* < 0.05 and heterogeneity evaluated using the I^2^ statistic. **Results**: A systematic search identified 90 records, of which 5 studies met the inclusion criteria. These studies, encompassing a total population of 4,427,291 children from developed countries, suggest a protective effect of rotavirus vaccination against T1D. The pooled HR was 0.87 (95% CI: 0.78–0.98), indicating a 13% lower risk of T1D in vaccinated children compared to unvaccinated ones (*p* = 0.03). Moderate heterogeneity was noted (χ^2^ = 10.02, df = 4, *p* = 0.04, I^2^ = 60%). **Conclusions**: This analysis suggests that rotavirus vaccination may reduce the risk of T1D in children from high-income Western countries. While these findings are promising, they may not be generalizable to settings outside similar advanced healthcare systems. Further research is needed to confirm the protective effects of rotavirus vaccination against T1D across diverse populations.

## 1. Introduction

Type 1 diabetes mellitus (T1D) is a long-term autoimmune condition that involves the destruction of beta cells in the pancreas that produce insulin, resulting in insulin deficiency. It primarily affects children and young adults, representing about 85% of diabetes cases in individuals under 20 years of age worldwide, with the highest incidence occurring between age 10 and 14 [1]. The rate of T1D has risen by about 70% in young children over the last two decades [2], and despite progress in the treatment options, the condition remains linked to substantial morbidity. Hypoglycemia and ketoacidosis are critical complications associated with T1D, while cardiovascular disease, retinopathy, diabetic renal disease, and neuropathy are the leading contributors to morbidity and mortality in individuals with T1D [3,4,5].

Before the onset of type 1 diabetes, there is a preclinical phase during which beta-cells maintain enough functionality to regulate blood glucose effectively. The time between the first laboratory appearance of antibodies and the clinical diagnosis of the disease can vary from less than a year to over two decades [6]. Thus, the progression of T1D can vary widely between individuals [6]. This variability highlights the importance of early detection and intervention strategies, since addressing the disease during the preclinical stage may offer a valuable opportunity to slow the progressive destruction of beta cells.

The pathophysiology of type 1 diabetes involves a complex interplay of genetic factors and environmental triggers, leading to the destruction of pancreatic beta cells by autoreactive T cells. Recent studies have increasingly focused on how environmental factors contribute to the development of T1D in genetically predisposed children [7]. Most studies investigating infectious triggers of T1D have focused on viral agents with viruses such as coxsackievirus B, influenza A, and herpeviruses being considered as significant environmental triggers for the disease [7,8,9]. The specific underlying mechanisms vary depending on the virus, but several hypotheses have emerged. These include molecular mimicry (where viral sequences resemble body proteins), infection-related alterations to the gut mucosa, direct infection of the pancreas, and interactions between the developing immune system and the timing of the infection [10]. Other factors, such as early exposure to cow’s milk proteins, vitamin D deficiency, maternal influences during pregnancy or early childhood, and environmental pollutants, have also been associated with an increased risk of developing T1D [7].

Vaccinations are also gaining attention for their potential impact on the onset of T1D. Evidence suggests that the inactivated influenza vaccine may reduce the risk of T1D in certain populations, such as Finnish children, with this effect potentially explained by the pleiotropic properties of the vaccine [9,11]. Additionally, much research has been conducted on the effect of the Bacillus Calmette–Guérin (BCG) vaccine in preventing T1D, following experimental studies that showed suppression of insulitis after BCG vaccination [12]. However, two recent meta-analyses found no protective effect of BCG vaccination on T1D development [13,14].

Rotavirus infection has, over the years, gained a lot of attention since it is the leading cause of severe gastroenteritis and dehydration in children under 5 years of age worldwide [15]. Rotaviruses are highly contagious and spread via the oral-fecal route, with nearly all children expected to be infected by the age of five [13]. In response to this global health burden, the development of rotavirus vaccines has been a critical area of research. The first licensed rotavirus vaccine, RotaShield, was introduced in 1998, but was withdrawn shortly after its release due to an increased risk of intussusception, a rare but serious bowel obstruction [16]. Since 2006, two vaccines, Rotarix™ (RV1) and RotaTeq™ (RV5), have been available worldwide. Rotarix™, a monovalent vaccine developed by GSK Biologicals in Belgium (Rixensart), is based on a single human rotavirus strain (G1P) [17]. It is given in two oral doses, spaced 4 weeks apart, to infants between 6 and 24 weeks of age [18]. RotaTeq™ developed by Merck & Co., Inc., Kenilworth, NJ, USA, is a pentavalent vaccine containing five human bovine reassortant strains (G1, G2, G3, G4, and P) [17]. It is administered in three oral doses, typically between 6 and 32 weeks of age [18]. The introduction of these effective vaccines has resulted in a marked reduction in related hospitalizations and mortality [19].

Apart from this significant reduction in related morbidity, studies have shown evidence that rotavirus vaccination may have a benefit in other syndromes such as childhood seizures, Kawasaki disease, and celiac disease [20,21,22,23]. The connection between the rotavirus vaccine and T1D has attracted attention in recent years, primarily due to the possibility that rotavirus infections may act as an environmental trigger for the disease [24]. Consequently, it has been suggested that the rotavirus vaccine may help in lowering the risk of T1D by preventing rotavirus infection [25]. However, the direct causal relationship and whether the rotavirus vaccination has a protective or non-protective action on the onset of T1D remains unclear [25].

This systematic review and meta-analysis aims to assess the impact of rotavirus vaccination on the development of T1D in childhood.

## 2. Methods

This systematic review and meta-analysis was conducted in accordance with the Preferred Reporting Items for Systematic Reviews and Meta-Analyses (PRISMA) 2020 guidelines [26]. The review protocol was registered prospectively with PROSPERO (CRD42024584739).

### 2.1. Search Strategy and Selection Criteria

We systematically searched PubMed, Google Scholar, Scopus, WHO—Global Index Medicus, DOAJ, and the Cochrane Library for studies published from 2006, when the two rotavirus vaccines became available globally, to the present, restricted to English language papers. The search terms included “rotavirus vaccination” and “type 1 diabetes”. To minimize potential geographical bias, we incorporated the WHO Global Index Medicus database into our research protocol. The GIM offers comprehensive access to the biomedical and public health literature from low- and middle-income countries worldwide, thereby enhancing the visibility and accessibility of these valuable resources.

We included randomized controlled trials (RCTs), cohort studies, and case–control studies that assessed children who were partially or fully vaccinated against rotavirus. Studies in languages other than English and studies involving adults and children diagnosed with T1D prior to partial or complete vaccination were excluded. Additionally, ecological studies, letters to the editor, case series, and systematic reviews were excluded.

### 2.2. Data Extraction and Quality Assessment

Two independent reviewers (A.K. and E.K.) conducted the title/abstract screening and full-text reviews in duplicate. Disagreements were resolved by a third reviewer (F.L.). The extracted data included study design, population size and characteristics, type of vaccine (RotaTeq or Rotarix), vaccination status, and effect estimates with corresponding confidence intervals. The ROBINS-E tool (https://www.riskofbias.info/, accessed on 30 August 2024) was used to assess the risk of bias, and only studies deemed to be low risk were included in the meta-analysis.

### 2.3. Statistical Analysis

Summary hazard ratios (HRs) were calculated by pooling study-specific estimates. Whenever available, adjusted effect estimates from individual studies were used. In cases where multiple adjusted effect estimates were provided for fully or partially vaccinated populations, the analysis focused on pooling the effect estimate comparing the reference population (unvaccinated children) to the largest intervention group (fully or partially vaccinated). Given the low incidence of T1D in the general population (approximately 15 per 100,000 individuals) [27], HRs were treated as approximations of incidence rate ratios (IRRs) where necessary. Weights were assigned to each study using the generic inverse variance method, and summary HRs were computed using a fixed-effects model. Whenever available, we used adjusted effect estimates from each study. Heterogeneity was evaluated using the I^2^ statistic, which ranges from 0% to 100%. All statistical analyses were performed using Review Manager (version 5.4.1), with statistical significance set at *p* < 0.05. Due to the limited number of studies, subgroup analyses based on vaccination status (fully vs. partially vaccinated) or study design (cohort vs. case–control vs. randomized controlled trial) could not be performed.

## 3. Results

### 3.1. Study Selection

**Figure 1:**
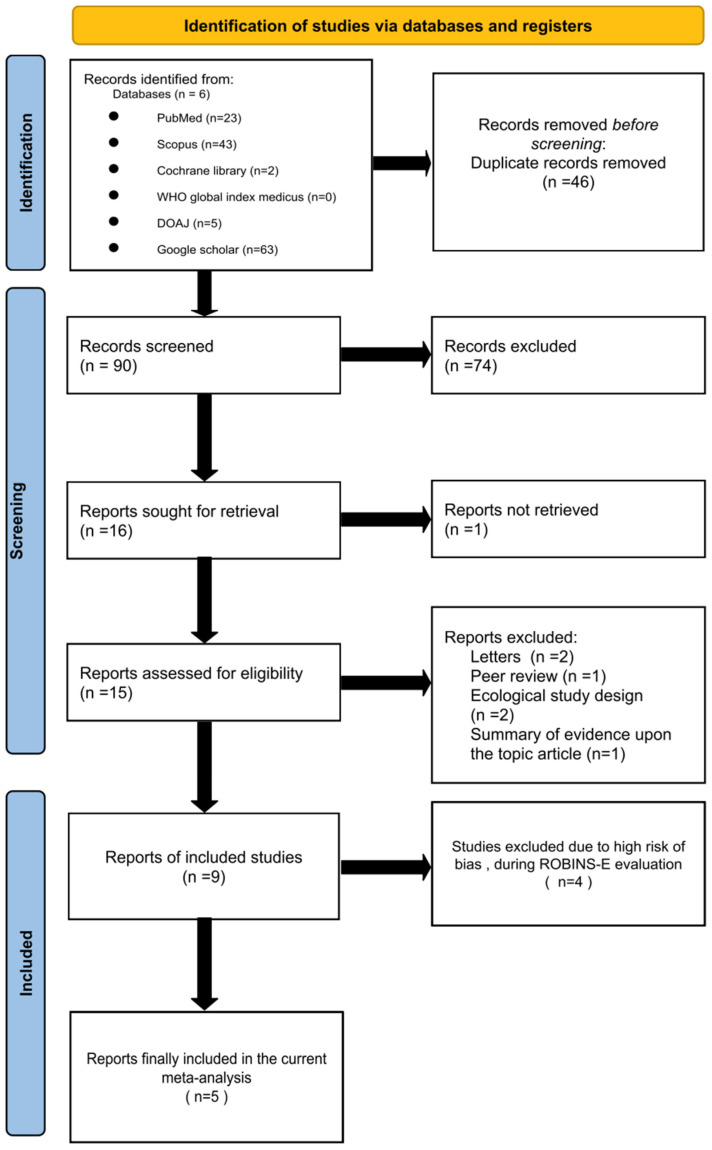
PRISMA 2020 flow diagram for new systematic reviews that included searches of databases and registers only.

After removing duplicates, 90 records were identified from database searches and 74 were excluded based on title and abstract screening. We retrieved fifteen full-text articles, of which nine studies met the inclusion criteria and were included in the systematic review. The PRISMA flow diagram (Figure 1) outlines the study selection process. We re-assessed all eligible studies for inclusion.

### 3.2. Included Studies

**Figure 2:**
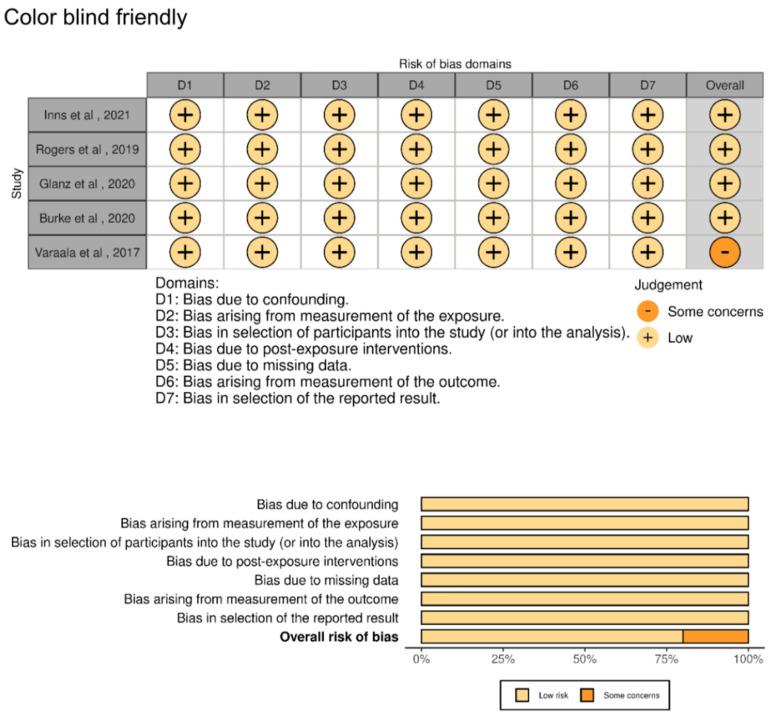
Risk of bias in the included studies. The five studies included in the analysis were considered to have a low risk of bias [10,25,28,29,30].

The included studies consisted of one RCT, three prospective cohort studies, one retrospective cohort study, one interrupted time series analysis, one nationwide registry study, and one population-based cohort study. Of the nine studies included, five were judged to have a low risk of bias (Figure 2) and four were rated as having a high risk due to concerns about confounding and selective reporting. The meta-analysis included data from the five studies with low risk of bias, representing a total population of 4,427,291 children (Table 1 and Table 2). Of the five studies included, four [10,25,28,29] reported HRs as their effect measure, while Vaarala et al. used IRRs [30].

### 3.3. Primary Outcomes

The meta-analysis demonstrates that rotavirus vaccination had a protective effect against the development of type 1 diabetes in children. The pooled hazard ratio was 0.87 (95% CI: 0.78 to 0.98), indicating a statistically significant 13% reduction in the risk of type 1 diabetes in vaccinated children compared to those that were unvaccinated (*p* = 0.03).

**Figure 3:**
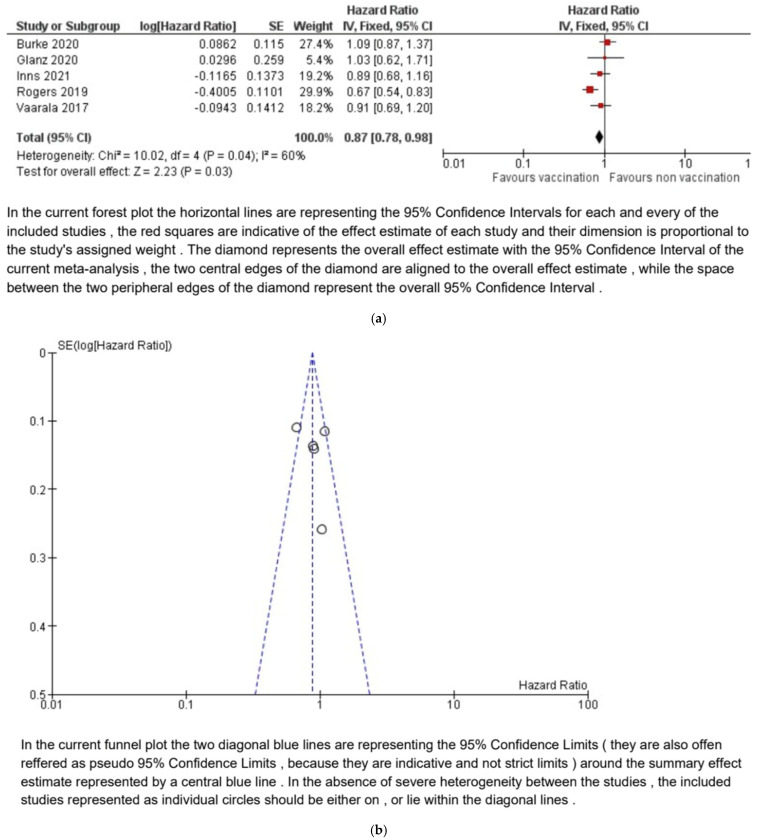
Forest (**a**) and funnel (**b**) plots of the meta-analysis [10,25,28,29,30].

Moderate heterogeneity was observed across the studies (χ^2^ = 10.02, df = 4, *p* = 0.04, I^2^ = 60%). Due to the limited number of studies, subgroup analyses (e.g., by vaccination status or study design) were not performed. Forest and funnel plots are presented in Figure 3.

## 4. Discussion

Research on the non-specific effects of live attenuated vaccines is growing [31,32], revealing benefits such as lower mortality rates that cannot be solely explained by protection against the targeted disease [33,34,35,36]. In contrast, there have been limited investigations into the non-specific effects of rotavirus vaccines, even though they have been widely used for over ten years. One study in the United States, however, found a reduction in hospitalizations for non-target diseases following rotavirus vaccination [25]. In the current meta-analysis of five studies, rotavirus vaccination appears to have a protective effect against the development of type 1 diabetes in children by reducing by 13% the risk of type 1 diabetes in vaccinated children.

A previous meta-analysis conducted by Zhang et al. in 2022, which included 5,793,055 children, found no protective effect of rotavirus vaccination against type 1 diabetes (RR 0.94, 95% CI: 0.82–1.09). The study also revealed no significant association between full or partial rotavirus vaccination and the risk of T1D. However, the analysis indicated that vaccinated children may have a lower risk of developing T1D before the age of 5 compared to those at age 5 or older, concluding that the effect of rotavirus vaccination on T1D risk may be time-dependent [37]. The authors attributed this finding to the possibility of a gradual waning of the rotavirus vaccine’s effectiveness over time or the fact that rotavirus infections predominantly affect children under 2 years of age, with the highest incidence occurring between 6 and 24 months. Consequently, the off-target effects are more likely to be observed in children under 5. However, it is important to note that this result had limited statistical power.

The results of this previous meta-analysis are in contrast with two articles published in 2019 and are included in the present systematic review. Perrett et al. performed an interrupted time-series analysis to assess the impact of the rotavirus vaccine on T1D incidence in Australia. They found a 15% decrease in T1D cases among children aged 0–4 years after the vaccine was introduced compared to before its introduction. However, no significant effect was observed in older age groups [38]. The authors found no evidence of an effect on incidence in older age groups [38]. Similarly, the cohort of Rogers et al., 1,474,535 infants, showed a 33% reduction in the risk of T1D in vaccinated children for rotavirus compared to unvaccinated children [25]. There was a total of a 3.4% decrease in the annual incidence of T1D with the vaccine introduction in 2006 in children 0–4 years old in the United States [25]. The authors of the study observed a decrease in the incidence of T1D among children who completed the full rotavirus vaccination series, but no reduction was found in those who only received part of the recommended doses. Additionally, they noted that the pentavalent vaccine, specifically, was linked to a lower risk of developing T1D.

The other studies included in the current review did not show a significant reduction in T1D incidence. In the study of Inns et al., rotavirus vaccination was not associated with the risk of T1D during a follow-up of 7 years. Their results were deemed generalizable to the broader population of England, as they were derived from the Clinical Practice Research Datalink Aurum. This database includes patients from General Practices across England, ensuring a representative sample of the population and providing detailed information for analysis [28]. The possible association of rotavirus infection and T1D was also investigated in a population-based cohort study with >120,000 children in Finland, where the incidence of T1D is the highest in the world. Data from the National Vaccination Register were used to categorize children born in 2009–2010 as either vaccinated (having received at least one dose) or unvaccinated against rotavirus. Information on T1D diagnoses was obtained from the National Care Register for the period 2009–2014. The authors calculated an adjusted relative risk of 0.91 (95% confidence interval [CI]: 0.69–1.20) for the association between rotavirus vaccination and T1D, indicating that vaccination does not significantly affect the risk of developing T1D during the first 4–6 years of life [30]. Another large cohort study based on commercial insurance data, published in January 2020, monitored children until they reached a maximum age of 12 years and found no association between rotavirus vaccination and T1D incidence [24].

In a retrospective cohort study, published in May 2020, including 386,937 children born between 2006 and 2014 and conducted in seven US health centers [29], the eligible children (aged from 8 months to 11 years) were divided into three groups according to their full exposure (93.1%), partial exposure (4.1%), or non-exposure (2.8%) to rotavirus vaccination and were followed-up for an average of 5.4 years. Out of the entire group, 464 children were diagnosed with type 1 diabetes, with an incidence rate of 20.6 cases per 100,000 person-years. Compared to the children who were unvaccinated, the adjusted hazard ratio (aHR) for developing diabetes was calculated to have no significant difference between the other subgroups of the study, children partially vaccinated (aHR: 1.50 (95% CI, 0.81–2.77)) and fully vaccinated with rotavirus (aHR: 1.03 (95% CI, 0.62–1.72)) [29].

It is important to note that the above studies were conducted with a defined follow-up period, which may have limited their ability to fully assess the long-term effects of rotavirus vaccination on T1D. It is possible that the vaccine provides a protective effect against the development of T1D later in life, but such an effect might not be observable within the shorter timeframes covered by these studies. Additionally, the incidence of T1D is relatively low in children in young age groups and increases with time, which further complicates the ability to detect any potential vaccine-related impact in this age group. Longer follow-up periods and studies involving older populations may be necessary to comprehensively evaluate this potential relationship.

In the present meta-analysis, due to the limited number of studies available, a subset analysis examining the effect of partial rotavirus vaccination on the incidence of T1D could not be conducted. Rogers et al. found no reduction in the incidence of T1D when there was only a partial vaccination against rotavirus [25]. The study of Inns et al. could not assess for partial vaccination effect since only 5.7% of participants received only one dose [28]. In the study of Burke et al., out of the total group of children who were followed-up (1,563,540), 66% were fully rotavirus vaccinated, 13% were partially vaccinated, and 20% were not vaccinated. Throughout the follow-up, the incidence of T1D was analyzed for each of the three groups. The hazard ratio was calculated showing no significant difference between the subgroups (fully vaccinated HR: 1.09 (0.87–1.36), partially vaccinated HR: 1.03 (0.78–1.36)) compared to unvaccinated children. Thus, the study showed no correlation between the components studied [24].

This analysis could not evaluate the association between the type of rotavirus vaccine and protection against T1D due to insufficient data. However, in the study by Rogers et al., the pentavalent RotaTeq vaccine demonstrated a greater protective effect against T1D compared to the monovalent Rotarix vaccine. Specifically, RotaTeq was associated with a 37% reduction in T1D risk, while Rotarix was linked to a 27% reduction [25]. Sensitivity analyses in the study by Rogers et al. revealed a significant association between rotavirus vaccination and several outcomes, including insulin use (HR = 0.71), hospitalization for T1D (HR = 0.70), and the occurrence of two or more T1D diagnoses along with insulin use (HR = 0.70). However, no reduction in incidence was observed in children who received only a partial vaccination series [25].

The first hypothesis that rotavirus vaccination may have an impact on T1D manifestation arose from experimental studies that showed that rotavirus accelerated pancreatic beta cell destruction in mice that were prone to diabetes since they had established insulitis [39]. Moreover, in children, the development of antibodies to rotavirus was linked to an increase in islet antibodies [40]. Various mechanisms have been suggested to explain how a rotavirus infection could trigger the onset of T1D [10]. Beyond direct pancreatic infection and beta-cell damage, studies have highlighted sequence similarities between rotavirus proteins and key autoantigens associated with T1D, such as islet-antigen 2 and glutamic acid decarboxylase 65 (GAD65) [41,42]. This resemblance may trigger molecular mimicry, where T cells activated against rotavirus cross-react with islet proteins, leading to autoimmunity [43,44,45]. Specifically, the viral protein VP7 of rotavirus may bind to human leukocyte antigen molecules linked T1D, triggering T-cell proliferative responses [40]. Another plausible mechanism involves bystander activation, where preexisting autoreactive T cells are inadvertently activated during the immune response to rotavirus infection [10,46]. Vaccination prevents rotavirus infection, thereby reducing the risk of such immune activation and its downstream effects.

The fixed-effects model was chosen in the present analysis due to the small number of included studies, the consistent use of the same rotavirus vaccines (RotaTeq and Rotarix), both of which have comparable effectiveness in the vaccination programs of the included studies [19], and the fact that these studies were conducted in countries with similarly advanced healthcare systems capable of reliant and consistent immunization against rotavirus and insurance services (both state and private) with reliable and digitalized health records [47]. As such, the included studies were considered functionally equivalent. While we consider the studies included in our analysis to be functionally equivalent, we recognize the influence of factors such as genetic diversity (both between countries of origin and within multiethnic populations in each country) and disparities in healthcare access due to social and income stratification, even in high-income countries. In terms of genetic diversity, vaccine manufacturers GSK and Merck ensured multiethnic representation in their clinical trial samples, demonstrating the consistent effectiveness of their products across diverse populations [48,49]. However, when it comes to healthcare access and insurance disparities, we acknowledge that the findings of this analysis should not be generalized beyond high-income countries or families with the financial means to meet basic healthcare needs. Specifically the findings of this meta-analysis cannot be safely generalized beyond the countries of the United States of America, the United Kingdom, and Finland. We also emphasize the importance of robust and consistent immunization programs in both developed and developing nations. Such efforts not only improve public health outcomes, but also contribute to a larger pool of reliable studies, enabling a more comprehensive understanding of the topic at hand.

The current data, as outlined in the introduction, clearly indicate that the incidence of type 1 diabetes is increasing. This trend does not align with the widespread implementation of rotavirus vaccination, which might be expected to contribute to a reduction in diabetes incidence. It is crucial to recognize that the pathophysiology of type 1 diabetes is multifactorial, with numerous environmental factors potentially contributing to the rising incidence despite extensive rotavirus vaccination coverage. Expanding vaccination programs in developing countries and assessing their impact on diabetes incidence in these regions could provide valuable insights into the vaccine’s efficacy in this context.

The strengths of our systematic review and meta-analysis include strict adherence to PRISMA and Cochrane guidelines, along with a large total sample size of children. Additionally, the risk of bias is minimized due to the exclusive inclusion of cohort studies, which are less susceptible to bias, as well as the selection of only studies with low risk of bias. The moderate level of heterogeneity further contributes to the reliability of the findings. Limitations of this study should also be addressed. For a low risk of bias, only five studies were included in this meta-analysis, since some retrieved studies had a high risk of bias, and thus were excluded from the current analysis. Furthermore, the limited number of studies did not allow for a subset analysis, for example, for vaccination status (full or partial) or testing for other factors that may influence the manifestation of T1D such as family history. Notably, all three U.S. studies exclusively utilized data from the largest commercial insurance databases, excluding smaller-scale databases. This approach limited the pool of potential participants, potentially introducing selection bias into the original studies. Moreover, it is important to note that the results should not be generalized beyond high-income Western countries with similarly advanced healthcare systems. Longer individual longitudinal studies, with long-term periods of follow-up, are necessary to evaluate whether the vaccine prevents the manifestation of T1D or just delays the manifestation of diabetes.

## 5. Conclusions

This meta-analysis found that rotavirus vaccination may reduce the incidence of type 1 diabetes in children. However, these findings may have been impacted by the possible introduction of selection bias in some of the original studies. Also, the above proved that the protective effect of rotavirus vaccination against T1D may not be applicable to low-income countries. Further longitudinal studies are needed to confirm the protective effects of rotavirus vaccination against T1D in diverse populations.

## Figures and Tables

**Table 1:** Table of characteristics of the finally included in the analysis studies.

Author	Journal and Publication Year	Country	Study Design	Vaccine	Population Under Study and Data Sources	Exposure Definition (Vaccination Status)	**Available Effect Estimate (95% CI)**
Inns et al. [28]	BMC Medicine, 2021	**United Kingdom**	Cohort	**Rotarix**	Children enrolled in CPRD (Clinical Practice Research Datalink) Aurum Born in the UK between 1 January 2010 and 31 December 2015 Registered at a CPRD GP practice within 14 weeks of birth Follow-up period began at 6 months of age (upper age limit for the second dose of Rotarix^®^ in the UK vaccination schedule)	Having a record of one or more doses of Rotarix in CPRD aurum **(fully or partially vaccinated in the same group)**	**Adjusted HR** 0.89 (0.68, 1.19)
Rogers et al. [25]	Scientific Reports, 2019	**United States of America**	Cohort	**RotaTeq or Rotarix**	Data from a nationwide health insurer in the United States (Clinformatics DataMart^®^ Database via OptumInsight, Eden Prairie, MN, USA) Data collection period: from 1 January 2001 to 30 June 2017 Infants < 1 year of age at the start of insurance coverage and continuous health insurance coverage for at least 365 days Infants were categorized into three groups: full vaccination, partial vaccination, and unvaccinated	Three exposure groups: Full rotavirus vaccination Partial vaccination (started but not completed) No vaccination **(Fully and partially vaccinated were in separate groups)**	**Adjusted HR** **(complete vaccination)** 0.67 (0.54, 0.83)
Glanz et al. [29]	JAMA Pediatrics, 2020	**United States of America**	Cohort	**RotaTeq or Rotarix**	Children enrolled in seven integrated healthcare organizations in the United States Participated in the Vaccine Safety Datalink (VSD) program Continuous health plan enrollment from 6 weeks to 2 years of age Born between 1 January 2006 and 31 December 2014	Three exposure groups: Full rotavirus vaccination Partial vaccination (started but not completed) No vaccination **(Fully and partially vaccinated were in separate groups)**	**Adjusted HR** **(complete vaccination)** 1.03 (0.62, 1.72) **Adjusted HR** **(partial vaccination)** 1.5 (0.81, 2.77)
Burke et al. [10]	JAMA Pediatrics, 2020	**United States of America**	Cohort	**RotaTeq or Rotarix**	Children continuously enrolled in their insurance plan from birth Eligible for rotavirus vaccination and born on or after 1 January 2006 Data collected from the IBM MarketScan Commercial Database Data span: from January 2006 to December 2017	Three exposure groups: Full rotavirus vaccination Partial vaccination (started but not completed) No vaccination **(Fully and partially vaccinated were in separate groups)**	**Adjusted HR** **(complete vaccination)** 1.09 (0.87, 1.36) **Adjusted HR** **(partial vaccination)** 1.03 (0.78, 1.36)
Varaala et al. [30]	The Pediatric Infectious Disease Journal, 2017	**Finland**	Cohort	**RotaTeq**	Children born in Finland between 2009 and 2010 Vaccination records retrieved from the National Vaccination Register (NVR) of Finland Data collected from 2009 to 2011 Children considered vaccinated after receiving their first vaccine dose	The children were considered vaccinated from the time of the first rotavirus vaccine dose **(fully or partially vaccinated in the same group)**	**IRR (adjusted)** 0.91 (0.69, 1.2)

**Table 2:** Population of the included studies.

**Study**	**Total Participation**	**Total Vaccinated Children** **(Partially or Fully)**		**Total Not Vaccinated Children**	**Type 1 Diabetes Cases in Vaccinated Children** **(Partially or Fully)**	**Type 1 Diabetes Cases in Non-Vaccinated Children**
Inns et al. [28]	880,629	343,113		537,516	240 (0.7‰)	493 (0.9‰)
Rogers et al. [25]	1,474,535	680,963		793,572	273 (0.4‰)	1000 (1.26‰)
Burke et al. [10]	1,563,540	1,245,255		318,285	592 (0.5‰)	192 (0.6‰)
Glanz et al. [29]	386,937	375,934		11,003	447 (1.2‰)	17 (1.5‰)
Varaala et al. [30]	121,650	27,213		94,437	243 (8.9‰)	102 (1‰)
Total	4,427,291	2,672,478		1,754,813	1795 (0.6‰)	1804 (1‰)
**Study**	**Total Fully Vaccinated Children**	**Total Partially Vaccinated Children**	**Total Not Vaccinated Children**	**Type 1 Diabetes Cases in Fully Vaccinated Children**	**Type 1 Diabetes Cases in Partially Vaccinated Children**	**Type 1 Diabetes in Non-Vaccinated Children**
Burke et al. [10]	1,035,198	210,057	318,285	492	100	192
Rogers et al. [25]	540,317	140,646	793,572	192	81	1000
Glanz et al. [29]	360,169	15,765	11,003	415	32	17

## Data Availability

All the research data used are included in the reference section.
